# Validation of Stockholm3 for prostate cancer detection in a swiss non-referral cohort: balancing biopsy reduction and diagnostic performance

**DOI:** 10.1007/s00345-026-06377-2

**Published:** 2026-03-25

**Authors:** Jonas Mudlagk, Odile Minder, Ashkan Mortezavi, Benjamin Bulling, Julian Cornelius, Nicolas Arnold, Beat Roth, Livio Mordasini, Nicola Giudici

**Affiliations:** 1 Department of Urology, University Hospital Bern, Freiburgstrasse 37, Bern, 3010 Switzerland; 2 Uroviva AG, Urologie-Praxis Sursee, Christoph-Schnyder-Strasse 2b, Sursee, 6210 Switzerland; 3 University Hospital Zürich, Rämistrasse 100, Zürich, 8091 Switzerland

**Keywords:** Stockholm3 test, SWOP score, PSA density, PSA, Prostate cancer, Risk stratification

## Abstract

**Purpose:**

Prostate cancer (PCa) diagnosis relies on PSA testing, but its low specificity leads to overdiagnosis. Stockholm3 may improve diagnostic accuracy. This study evaluated the performance of Stockholm3 for detecting clinically significant PCa (csPCa), defined as ISUP grade ≥ 2 on biopsy, in a real-world, non-referral Swiss cohort.

**Methods:**

Retrospective analysis of a prospective registry (2023–2025) at a non-referral urology practice in Switzerland. Initial screening was performed in men with PSA > 1.5 ng/mL or a suspicious digital rectal examination. Patients who underwent Stockholm3 and biopsy were included. We compared Stockholm3 with PSA, PSA density, and the SWOP risk calculator for the detection of csPCa in a pre-MRI diagnostic setting.

**Results:**

We included 178 men (median age 65 years, IQR: 60.0–70.0). Among them, 60/178 (33.7%) had csPCa. Stockholm3 ≥ 15 showed sensitivity 0.78 (95% CI 0.67–0.88), specificity 0.44 (95% CI 0.35–0.54), and AUC 0.68 (95% CI 0.60–0.77). SWOP score > 4% showed sensitivity 0.42 (95% CI 0.30–0.55), specificity 0.78 (95% CI 0.70–0.85), and AUC 0.68 (95% CI 0.59–0.76). Both tests outperformed PSA > 3 ng/mL and PSA density > 0.15 ng/mL², with AUCs of 0.52 (95% CI 0.48–0.56) and 0.57 (95% CI 0.50–0.64), respectively. Decision curve analysis and calibration curves favored Stockholm3 over SWOP.

**Conclusions:**

In a non-referral setting, Stockholm3 confirmed good diagnostic performance for csPCa. These findings support its use as part of the primary diagnostic work-up in similar clinical settings.

## Introduction

Prostate cancer (PCa) remains one of the most commonly diagnosed cancers among men worldwide [1]. Early detection is crucial, but screening methods based on prostate-specific antigen (PSA) testing are limited by low specificity, leading to high rates of unnecessary biopsies and overdiagnosis of indolent cancers [2, 3].

In this context, alternative approaches that can be combined with standard diagnostic tools are essential. Their integration may allow for better patient stratification and help optimize the diagnostic pathway, reducing the number of unnecessary invasive procedures while maintaining high sensitivity to avoid missing clinically significant disease.

The blood-based Stockholm3 test has been proposed as an improved diagnostic alternative. It combines clinical variables, blood biomarkers, and genetic markers into a single risk score [4]. Its effectiveness has been validated across diverse populations and countries, demonstrating its accuracy and ability to reduce reliance on unnecessary Magnetic Resonance Imaging (MRI) and prostate biopsies [5–8].

The Rotterdam Prostate Cancer Risk Calculator (RPCRC, also known as the SWOP score) is another widely used risk stratification tool [9, 10]. It incorporates PSA, prostate volume, previous biopsy results, digital rectal examination (DRE) with optional MRI findings, to estimate an individual’s risk of prostate cancer, including clinically significant disease. Multiple studies have confirmed its utility in guiding biopsy decisions and improving the balance between cancer detection and reduction of unnecessary procedures [11–13].

However, studies validating risk calculators in this setting often originate from academic or referral centers, where the pretest probability is presumably higher and subjective parameters, such as DRE, are typically assessed by experienced high-volume physicians [14]. Evidence on their performance in non-referral, community-based settings is limited, yet crucial for broader implementation. This study evaluates the diagnostic accuracy of Stockholm3 in a Swiss non-referral cohort.

## Methods

### Patient population

Data from men with suspicion of prostate cancer who underwent the Stockholm3 test were collected in a prospective registry between November 2023 and January 2025 at a non-referral urology practice with multiple sites in Switzerland. Initial screening was performed in men with PSA > 1.5 ng/mL or a suspicious digital rectal examination (DRE). The decision to use Stockholm3 alongside the standard diagnostic work-up was made individually by the treating urologist. For this retrospective analysis, we extracted data from 178 patients who underwent PSA, Stockholm3 testing and prostate biopsy. Men with a prior diagnosis of prostate cancer were excluded. Clinical, imaging, and histopathological data were prospectively collected, including: age, family history, DRE findings, 5-ARI use, prior biopsy history, prostate volume, PSA, PSA density, Stockholm3 score, MRI findings (if available) with PI-RADS score and biopsy results. The study was conducted in accordance with ethical standards laid down in the 1964 Declaration of Helsinki and its later amendments. The study protocol was approved by the Cantonal Ethics Committee of Zurich (Kantonale Ethikkommission Zürich), Switzerland (BASEC-Nr. Req-2025-00421). All patients provided their consent by signing the Swiss General Consent form.

### Outcome definition

The primary outcome was clinically significant prostate cancer, defined as ISUP (International Society of Urological Pathology) grade ≥ 2 on biopsy.

### Prostate biopsy technique

All prostate biopsies were performed transrectally under local anesthesia. For patients with MRI lesions scored PI-RADS ≥ 3, three targeted cores were obtained from each lesion. All patients underwent additionally a systematic 12-core template biopsy.

### Index tests

The primary aim of this study was to compare the performance of the Stockholm3 test and the SWOP risk score without incorporating MRI data. Although MRI data were available, they were deliberately excluded from the calculation in order to simulate a clinical scenario in which MRI has not yet been performed, and the treating urologist must decide whether to proceed with MRI and subsequent biopsy based solely on initial risk stratification. The SWOP score was calculated using the official online tool (https://www.prostatecancer-riskcalculator.com), while the Stockholm3 test was performed at either the A3P Biomedical laboratory in Uppsala, Sweden, or the Labor Team laboratory in Goldach, Switzerland. The Stockholm3 test is a multivariable blood-based risk prediction model for csPCa that combines clinical variables (age, first-degree family history of PCa (father, brother or son), and previous biopsy history), blood biomarkers (total PSA, free PSA, free/total PSA ratio, hK2, MIC1, and MSMB), and genetic markers (a polygenic risk score based on 254 single-nucleotide polymorphisms [SNPs] and a specific variable for the HOXB13 SNP). The Rotterdam Prostate Cancer Risk Calculator (RPCRC), also known as the SWOP risk calculator, is a multivariable model derived from the European Randomized Study of Screening for Prostate Cancer (ERSPC). It incorporates PSA level, prostate volume, digital rectal examination findings, prior biopsy status, and optionally MRI findings to estimate an individual’s risk of csPCa. We applied the recommended 4% cutoff for the ERSPC/SWOP risk calculator, which has been externally validated in multiple cohorts [10, 12, 15]. As a secondary analysis, we assessed the performance of PSA (> 3 ng/mL) and PSA density (> 0.15 ng/mL²), using recommended clinical cutoffs, in the same pre-MRI setting for the detection of csPCa [16].

### Statistical analysis

We assessed the performance of Stockholm3 and the SWOP score across several dimensions: discrimination, calibration, clinical utility, and risk distribution. Discrimination of csPCa was evaluated by calculating the area under the receiver operating characteristic curve (AUC) and generating ROC curves for comparison. Calibration, defined as the agreement between predicted and observed outcomes, was assessed graphically. Clinical net benefit was evaluated using decision curve analysis (DCA) across a range of clinically relevant risk thresholds for prostate biopsy (0–0.5). We also computed sensitivity, specificity, positive predictive value (PPV), and negative predictive value (NPV) for each test. No missing values were present for the variables included in the analyses, as only patients with complete information for all required predictors were included in the study; therefore, a complete-case analysis was performed. For relative proportions, we report 95% confidence intervals that were calculated using 1000 bootstrap samples. All analyses were performed using R (version 4.5.1).

## Results

### Patient population

A total of 178 patients were included in the analysis. Their baseline characteristics are summarized in Table 1. The median age was 65 years (IQR 60–70). Seventeen patients (9.6%) had a prior negative biopsy, and 30/178 (19.2%) reported a positive family history of prostate cancer. The median PSA level was 5.20 ng/mL (IQR 4.13–7.01). A digital rectal examination (DRE) was performed in 79/178 patients (44%), of whom 5 (6%) had abnormal findings. Among the 165 men who underwent MRI, PI-RADS scores were distributed as follows: PI-RADS 1–2 in 25 patients (15.2%), PI-RADS 3 in 52 (31.5%), PI-RADS 4 in 66 (40.0%), and PI-RADS 5 in 22 (13.3%). The median Stockholm3 score was 17.0 (IQR 13.0–26.0). The SWOP score had a median of 2.4% (IQR 1.4–4.3). Regarding biopsy outcomes, 94/178 patients (53%) had benign results, and 24/178 (14%) were diagnosed with ISUP grade 1. csPCa (ISUP ≥ 2) was detected in 60/178 patients (34%), including 32/178 (18%) with ISUP 2, 17/178 (10%) with ISUP 3, 8/178 (5%) with ISUP 4, and 3 (2%) with ISUP 5 disease.

**Table 1:** Patient characteristics (*N* = 178)

Variable	Value
Age, median (IQR)	65.0 (60.0–70.0)
Prior negative biopsy, *n* (%)	17 (9.6%)
Family history positive, *n* (%)	30 (19.2%)
PSA, median (IQR)	5.20 (4.13–7.01)
Stockholm3, median (IQR)	17.0 (13.0–26.0)
SWOP score, median (IQR)	2.4 (1.4–4.3)
DRE Performed, *n* (%)	79 (44.4%)
Abnormal DRE, *n* (%)	5 (6.3%)
Prostate volume, median (IQR)	51.0 (40.0–70.0)
PI-RADS 1–2, *n* (%)	25 (15.2%)
PI-RADS 3, *n* (%)	52 (31.5%)
PI-RADS 4, *n* (%)	66 (40.0%)
PI-RADS 5, *n* (%)	22 (13.3%)
ISUP ≥ 2, *n* (%)	60 (33.7%)
ISUP 0 (Benign), *n* (%)	94 (52.8%)
ISUP 1, *n* (%)	24 (13.5%)
ISUP 2, *n* (%)	32 (18.0%)
ISUP 3, *n* (%)	17 (9.6%)
ISUP 4, *n* (%)	8 (4.5%)
ISUP 5, *n* (%)	3 (1.7%)

### Diagnostic metrics for csPCa

**Fig. 1:**
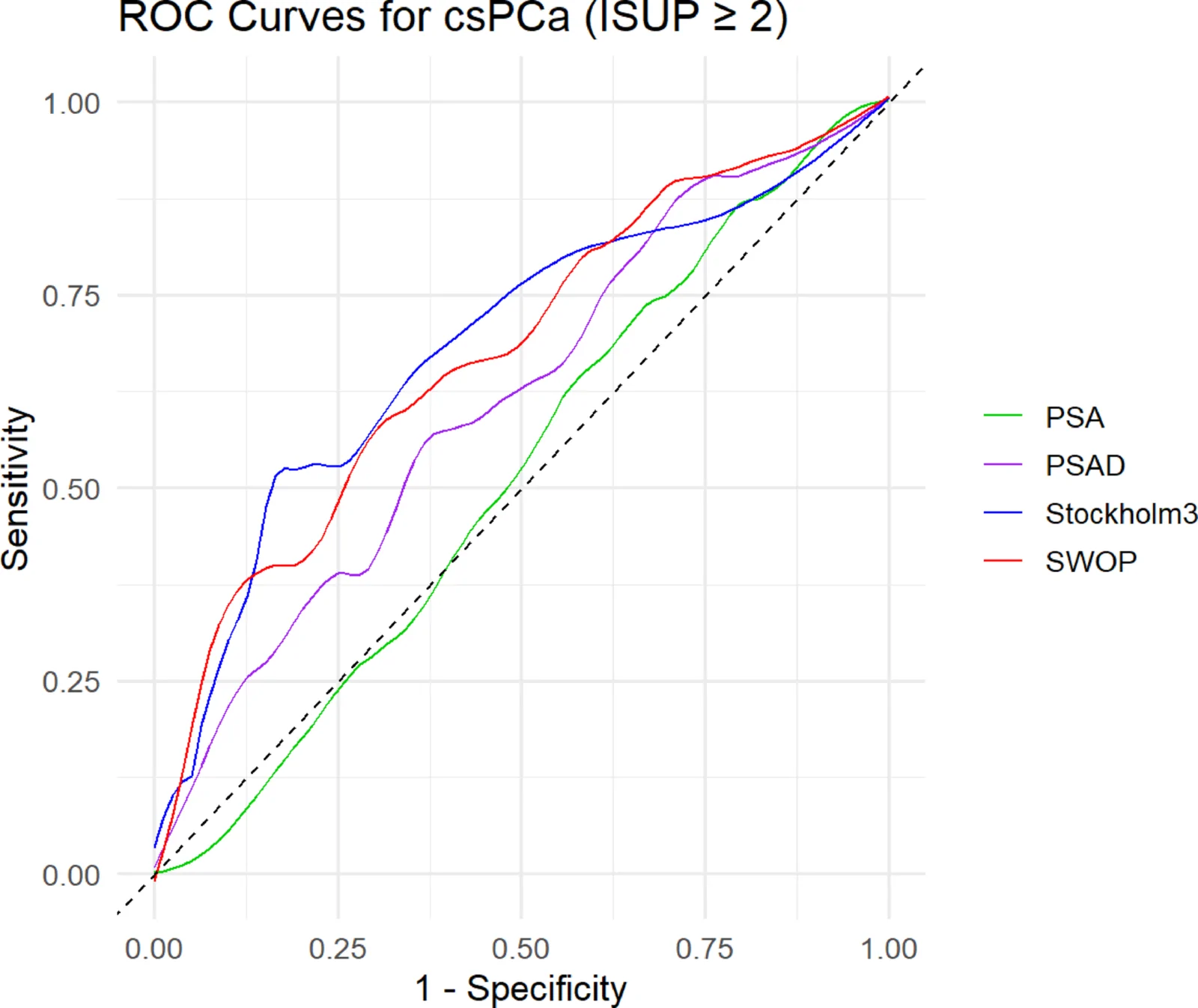
Receiver operating characteristic (ROC) curves showing sensitivity versus 1 – specificity for each model, along with the corresponding area under the curve (AUC) values

We evaluated the diagnostic performance of Stockholm3 at the ≥ 15 threshold and compared it with the SWOP score > 4%. Stockholm3 ≥ 15 demonstrated a sensitivity of 0.78 (95% CI 0.67–0.88), specificity of 0.44 (95% CI 0.35–0.54), and an AUC of 0.68 (95% CI 0.60–0.77). In comparison, the SWOP score > 4% achieved a sensitivity of 0.42 (95% CI 0.30–0.55), specificity of 0.78 (95% CI 0.70–0.85), and an AUC of 0.68 (95% CI 0.59–0.76), showing similar overall discriminative performance. We also compared the diagnostic accuracy of PSA and PSA density using their established cutoffs. PSA > 3 ng/mL showed high sensitivity [0.93 (95% CI 0.86–0.99)] but very low specificity [0.11 (95% CI 0.05–0.17)], leading to a high rate of false positives and an AUC of 0.52 (95% CI 0.48–0.56). In contrast, PSA density > 0.15 ng/mL² demonstrated higher specificity [0.81 (95% CI 0.74–0.88)] but lower sensitivity [0.32 (95% CI 0.21–0.44)], resulting in an AUC of 0.57 (95% CI 0.50–0.64). Table 2 summarizes the diagnostic performance metrics of the evaluated tests, while Fig. 1 presents the corresponding receiver operating characteristic (ROC) curves.

**Table 2:** Diagnostic metrics for clinically significant prostate cancer

Biomarker/test	Sensitivity (95% CI)	Specificity (95% CI)	PPV (95% CI)	NPV (95% CI)	AUC (95% CI)
Stockholm3 ≥ 15	0.78 (0.67–0.88)	0.44 (0.35–0.54)	0.42 (0.33–0.51)	0.80 (0.70–0.89)	0.68 (0.60–0.77)
SWOP > 4%	0.42 (0.30–0.55)	0.78 (0.70–0.85)	0.49 (0.35–0.63)	0.72 (0.65–0.80)	0.68 (0.59–0.76)
PSA > 3 ng/mL	0.93 (0.86–0.99)	0.11 (0.05–0.17)	0.35 (0.27–0.42)	0.76 (0.54–0.95)	0.52 (0.48–0.56)
PSA density > 0.15 ng/mL/cm³	0.32 (0.21–0.44)	0.81 (0.74–0.88)	0.46 (0.31–0.62)	0.70 (0.62–0.78)	0.57 (0.50–0.64)

### Potentially spared biopsies

**Fig. 2:**
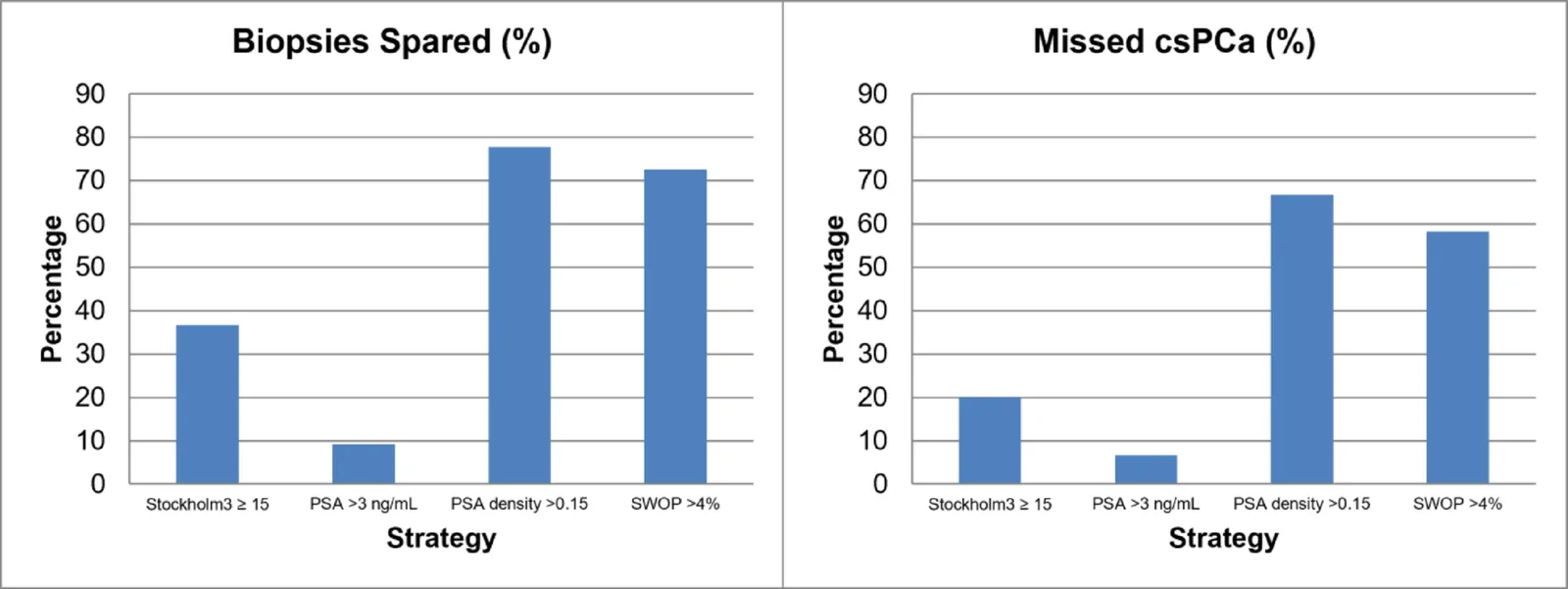
Comparison of biopsy-sparing and missed csPCa rates across diagnostic strategies

Applying these thresholds to our cohort, Stockholm3 ≥ 15 would have spared 36.5% of biopsies while missing 20.0% of csPCa cases. In comparison, PSA density > 0.15 ng/mL² and SWOP score > 4% would have spared 77.0% and 71.3% of biopsies, respectively, but at the cost of missing 66.7% and 58.3% of csPCa cases. Conversely, PSA > 3 ng/mL would have spared only 9.6% of biopsies while missing 6.7% of csPCa. Table 3; Fig. 2 summarize the proportions of potentially spared biopsies and missed clinically significant prostate cancers across the different diagnostic tests using the recommended cutoffs.

**Table 3:** Biopsy-sparing results table

Strategy	Biopsies spared *n* (%)	Missed csPCa *n* (%)
Stockholm3 ≥ 15	65 (36.5%)	12 (20.0%)
PSA > 3 ng/mL	17 (9.6%)	4 (6.7%)
PSA density > 0.15 ng/mL/cm³	137 (77.0%)	40 (66.7%)
SWOP > 4%	127 (71.3%)	35 (58.3%)

### Number needed to biopsy to detect one clinically significant prostate cancer

Using a Stockholm3 cutoff of ≥ 15, a total of 114 biopsies would have been performed, detecting 48 csPCa cases, corresponding to a Number Needed to Biopsy (NNB) of 2.38. In comparison, PSA > 3 ng/mL resulted in a NNB of 2.88, while PSA density > 0.15 ng/mL/cm³ yielded a NNB of 2.30, and the SWOP score > 4% a NNB of 2.04. Although PSA density and SWOP appeared more efficient in terms of biopsies per detected csPCa, these strategies missed a substantially higher proportion of csPCa.

### Potentially missed cancers

To further characterize the clinical implications of these thresholds, we examined the distribution of cancers that would have been missed. With a Stockholm3 cutoff of < 15, 12 csPCa cases would have remained undetected (6 ISUP 2, 3 ISUP 3, 2 ISUP 4, and 1 ISUP 5). In contrast, a SWOP threshold of ≤ 3% would have missed 35 csPCa cases, predominantly ISUP 2 (*n* = 24), but also higher-grade tumors (6 ISUP 3, 3 ISUP 4, and 2 ISUP 5). These findings demonstrate that Stockholm3 misses fewer csPCa than the SWOP Score, underscoring the limited sensitivity of SWOP at low-risk cutoffs in this pre-MRI diagnostic setting.

### Decision and calibration curves

**Fig. 3:**
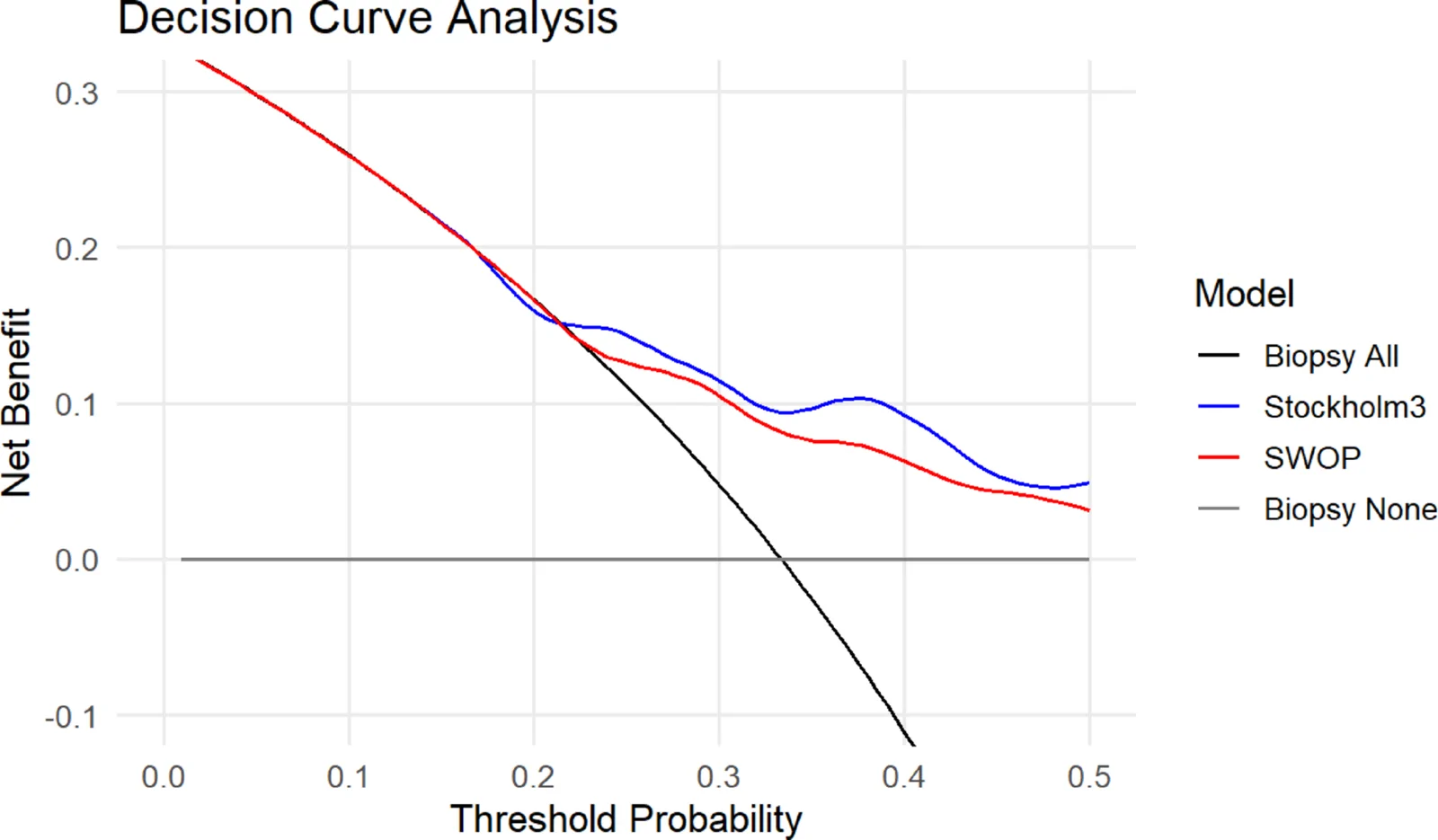
Decision curve analysis illustrating the net benefit for predicting csPCa on biopsy, with reference lines representing the biopsy-all strategy and the biopsy-none strategy

Net benefits were assessed using decision curve analysis (DCA) and compared with the strategies of biopsying all men and biopsying none, across risk thresholds from 0 to 0.5 for both the SWOP score and Stockholm3. Stockholm3 demonstrated a positive net benefit from a threshold probability of 21%, whereas the SWOP score showed a slightly lower net benefit from a threshold probability of 22%. Across the entire range of thresholds from 0 to 0.5, Stockholm3 slightly outperformed the SWOP score (Fig. 3).

**Fig. 4:**
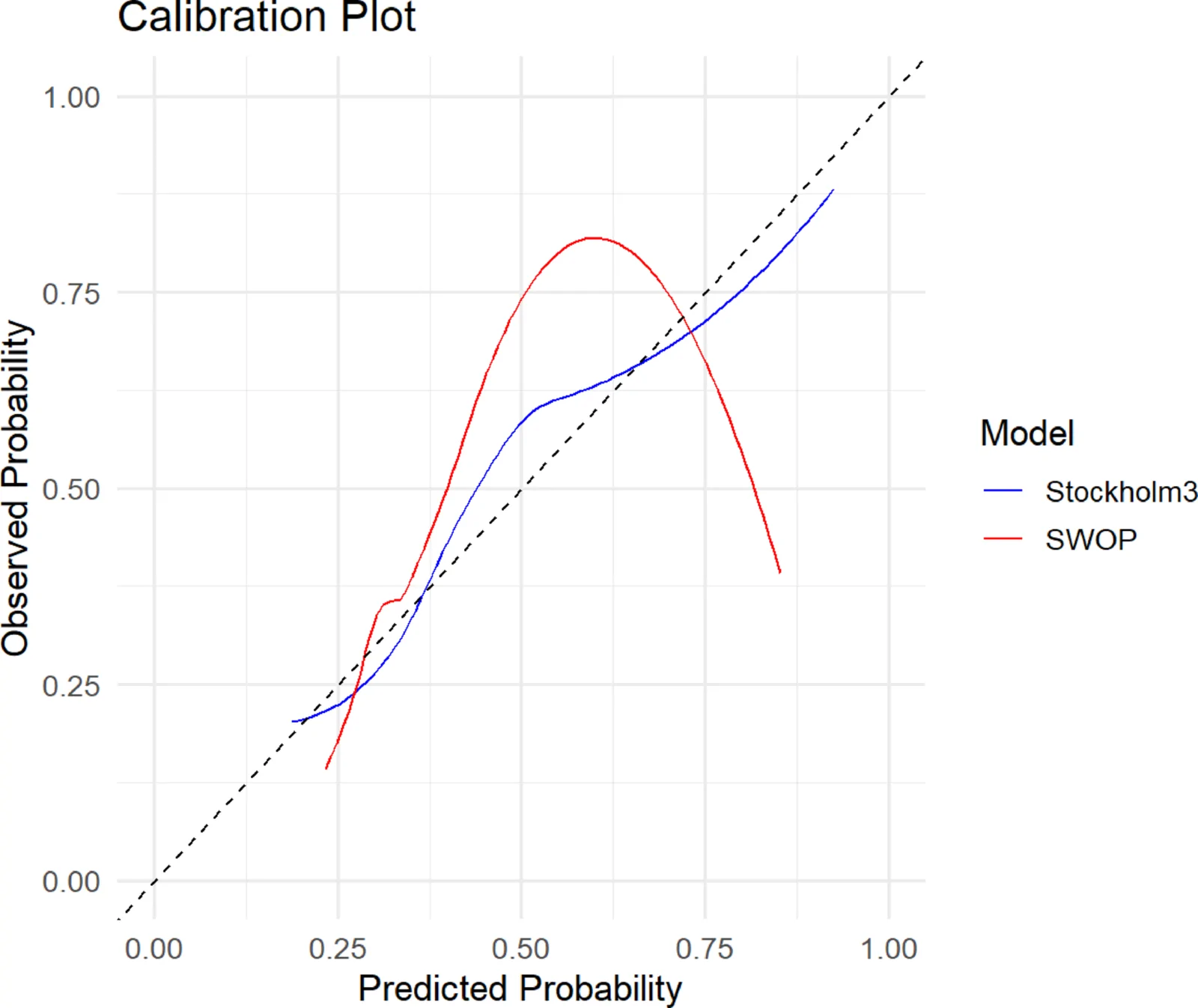
Calibration (observed probability vs. predicted probability) for risk prediction

Calibration was plotted for both the SWOP score and Stockholm3 (Fig. 4). Stockholm3 showed adequate calibration, whereas the SWOP score underestimated the observed risk, particularly for predicted probabilities above 30%.

**Fig. 5:**
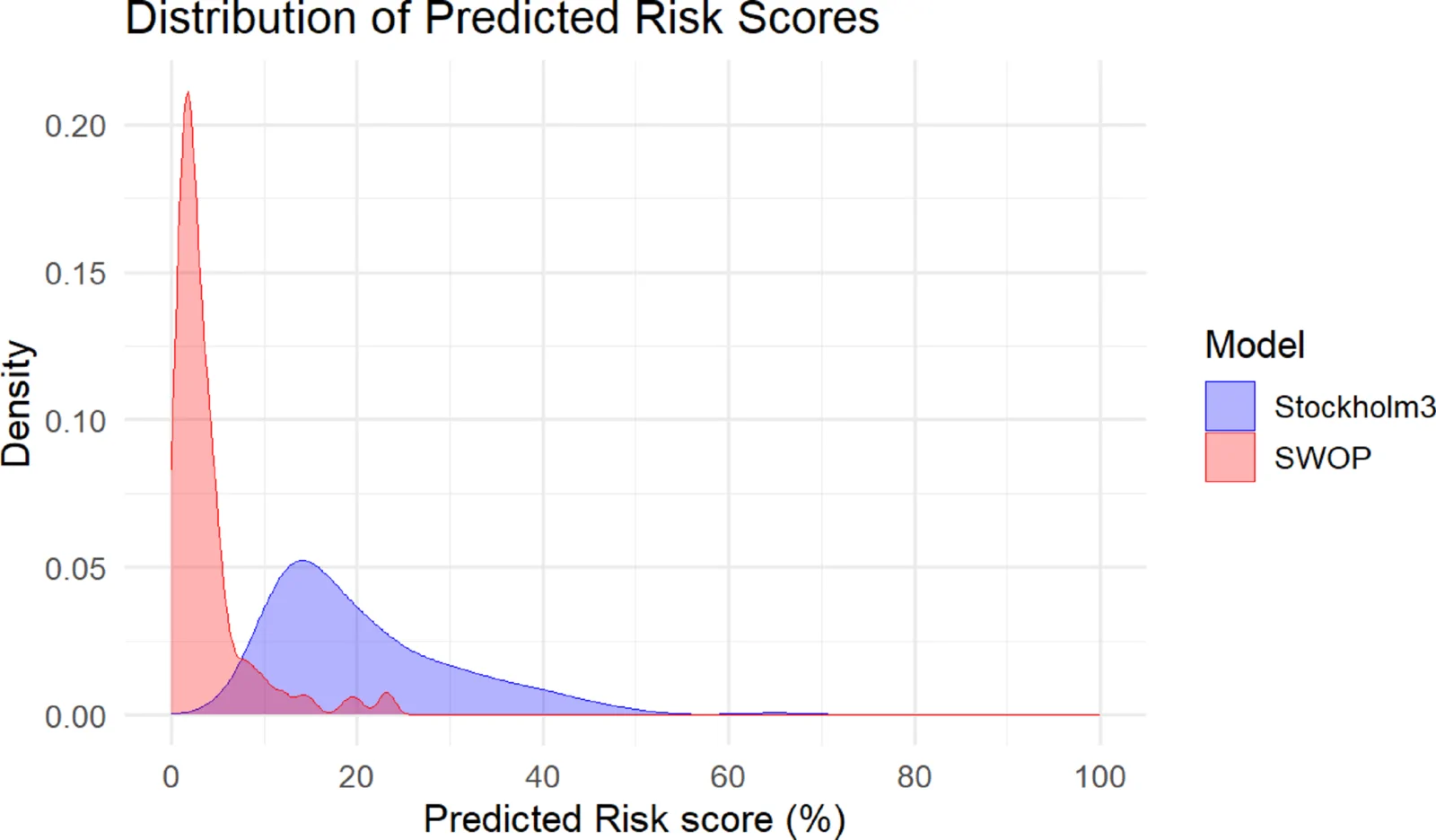
Distribution plot: The shaded areas represent the distribution of predicted risks for each risk calculator

In the distribution plot, the SWOP score shows a very narrow distribution from 0% to around 10%, which is consistent with the recommendation to use a 4% threshold (Fig. 5).

## Discussion

In this analysis of a prospective registry from a non-referral, community-based Swiss urology practice, we assessed the diagnostic performance of Stockholm3 in men evaluated for suspected PCa. To our knowledge, this is the first study conducted exclusively in a non-referral setting consisting of community-based urological practices. To enhance real-world relevance, we compared Stockholm3 against other diagnostic tests and their clinically established thresholds.

At the ≥ 15 threshold, Stockholm3 achieved an AUC of 0.68 (95% CI 0.60–0.77), comparable to the SWOP score > 4% (AUC 0.68, 95% CI 0.59–0.76). This threshold would spare 36.5% of biopsies while missing 20% of csPCa. PSA density (> 0.15 ng/mL²) outperformed PSA overall, but its high specificity and limited sensitivity suggest that lowering the threshold may be warranted in this clinical setting, as supported by other validation studies [17, 18]. Decision curve and calibration analyses confirmed the better performance of Stockholm3 compared with the SWOP score, in line with findings from previous studies [8, 11]. Importantly, unlike PSA density and the SWOP score, Stockholm3 is not dependent on prostate volume measurements or DRE, both of which often vary in reliability and availability across practices. This independence represents a key advantage of Stockholm3, particularly in non-referral settings, where interobserver variability and experience-dependent differences are well-recognized challenges [19, 20].

Our findings align with prior evaluations of Stockholm3 in the primary diagnostic setting. The original STHLM3 trial, conducted in Sweden among more than 58,000 men, demonstrated that the test improved discrimination over PSA, with an AUC of 0.74 compared to 0.56 for PSA, and could reduce unnecessary biopsies by 32% while maintaining detection of csPCa [4]. Subsequent external validations in North America and Europe reported AUC values ranging from 0.76 to 0.82, with biopsy reductions of 21–45% at comparable sensitivity levels [6, 21]. A recent German study further confirmed these results, showing that Stockholm3 achieved an AUC of 0.8, while avoiding around half of biopsies and missing fewer than 10% of csPCa cases [8]. While the absolute discrimination in our cohort appears somewhat lower than in other population-based studies, the relative advantage over PSA and the more balanced trade-off compared with PSA density or SWOP is consistent with prior evidence. Differences may in part reflect the relatively small sample size of our cohort, which may lead to less stable estimates of diagnostic performance. In addition, our study was conducted in a community-based, non-referral setting that reflects routine clinical practice, where variability in MRI interpretation, biopsy technique, and operator experience may influence diagnostic performance. Notably, the prevalence of clinically significant prostate cancer in our cohort (34%) was comparable to that reported in other contemporary external validation studies [8, 11]. Nevertheless, our findings reinforce the robustness of Stockholm3 across different healthcare systems and patient populations. However, further validation in larger, prospective cohorts is warranted, particularly to evaluate whether a slightly lower cutoff might optimize sensitivity without substantially increasing unnecessary biopsies.

In summary, our community-based Swiss cohort study extends the available evidence and has several practical implications. First, we showed that Stockholm3 maintains reliable performance even in lower-volume, non-specialist environments, underscoring its utility where MRI and biopsy quality may vary and expert interpretation is not always available. By providing a robust pre-imaging risk stratification that is independent of radiologist or urologist experience, Stockholm3 offers particular value for general urologists working in community practices or regions with limited access to high-quality imaging. Second, applying it could substantially reduce unnecessary MRIs and biopsies while maintaining clinically significant cancer detection, supporting a risk-adapted, individualized diagnostic pathway that moves beyond PSA-based screening. Finally, the practical implementation of Stockholm3 further supports its role as a scalable diagnostic tool. It can be seamlessly integrated into routine practice via an online platform, requiring only a blood draw and basic clinical information, thereby offering a low-barrier, widely applicable solution for early risk assessment.

Our study has several limitations. First, the retrospective design introduces potential selection bias, as testing indications were not standardized. In addition, verification bias may be present because only patients who ultimately underwent prostate biopsy were included in the analysis. Consequently, men considered at lower clinical risk may not have proceeded to biopsy, which could influence estimates of sensitivity, specificity, and overall diagnostic accuracy. However, data collection was prospective, which minimizes reporting bias, and this setting reflects real-world clinical practice, increasing the external validity of our findings by capturing genuine decision-making processes in community care.

Second, while we collected data on MRI and histopathology, we did not centralize MRI reading or biopsy interpretation. Thus, biopsy accuracy may vary depending on operator experience. This limitation, however, strengthens the generalizability of our findings to typical non-academic environments.

Third, we used the SWOP score without incorporating MRI input. While this was intentional to reflect the pre-imaging setting, it may underestimate SWOP score potential in combination with imaging. Nonetheless, this comparison is clinically relevant, as it simulates the decision point before MRI is considered. Fourth, the cost of the Stockholm3 test in Switzerland, approximately CHF 664 and currently borne by the patient, should be considered when evaluating its clinical implementation. In contrast, multiparametric prostate MRI, costing around CHF 1070, is reimbursed by health insurance. This difference in reimbursement policies may influence clinical decision-making and access to testing. The economic implications of implementing Stockholm3 therefore represent an important consideration at a national healthcare policy level. Although this study did not include a formal cost-effectiveness analysis, economic considerations will be important when assessing the broader feasibility and potential integration of Stockholm3 into routine clinical pathways. Lastly, our analysis was limited to a single urology practice, and no long-term outcomes were assessed. This may restrict the generalizability of our findings to other healthcare systems or populations and prevents evaluation of the test’s impact on treatment decisions, costs, and oncological outcomes. Further studies, ideally prospective, are needed to assess the performance and utility of the test in this setting.

## Conclusion

In this study conducted in community-based urology practices, Stockholm3 demonstrated promising overall performance for identifying men at risk of clinically significant prostate cancer in a non-referral setting. These results suggest that risk prediction with Stockholm3 could help refine diagnostic pathways and reduce unnecessary biopsies in everyday clinical practice. Further prospective validation is needed to confirm these results and determine the most appropriate cutoff for clinical practice.

## Data Availability

Requests for access to deidentified data may be considered upon reasonable request to the corresponding author.
